# The Nutritional Balancing Act of a Large Herbivore: An Experiment with Captive Moose (*Alces alces* L)

**DOI:** 10.1371/journal.pone.0150870

**Published:** 2016-03-17

**Authors:** Annika M. Felton, Adam Felton, David Raubenheimer, Stephen J. Simpson, Sophie J. Krizsan, Per-Ola Hedwall, Caroline Stolter

**Affiliations:** 1 Southern Swedish Forest Research Centre, Swedish University of Agricultural Sciences, Alnarp, Sweden; 2 School of Biological Science and The Charles Perkins Centre, the University of Sydney, Sydney, New South Wales, Australia; 3 Faculty of Veterinary Science, University of Sydney, Sydney, New South Wales, Australia; 4 Department of Agricultural Research for Northern Sweden, Swedish University of Agricultural Sciences, Umeå, Sweden; 5 Department of Animal Ecology and Conservation, Biocenter Grindel, University of Hamburg, Hamburg, Germany; Michigan Technological University, UNITED STATES

## Abstract

The nutrient balancing hypothesis proposes that, when sufficient food is available, the primary goal of animal diet selection is to obtain a nutritionally balanced diet. This hypothesis can be tested using the Geometric Framework for nutrition (GF). The GF enables researchers to study patterns of nutrient intake (e.g. macronutrients; protein, carbohydrates, fat), interactions between the different nutrients, and how an animal resolves the potential conflict between over-eating one or more nutrients and under-eating others during periods of dietary imbalance. Using the moose (*Alces alces* L.), a model species in the development of herbivore foraging theory, we conducted a feeding experiment guided by the GF, combining continuous observations of six captive moose with analysis of the macronutritional composition of foods. We identified the moose’s self-selected macronutrient target by allowing them to compose a diet by mixing two nutritionally complementary pellet types plus limited access to *Salix* browse. Such periods of free choice were intermixed with periods when they were restricted to one of the two pellet types plus *Salix* browse. Our observations of food intake by moose given free choice lend support to the nutrient balancing hypothesis, as the moose combined the foods in specific proportions that provided a particular ratio and amount of macronutrients. When restricted to either of two diets comprising a single pellet type, the moose i) maintained a relatively stable intake of non-protein energy while allowing protein intakes to vary with food composition, and ii) increased their intake of the food item that most closely resembled the self-selected macronutrient intake from the free choice periods, namely *Salix* browse. We place our results in the context of the nutritional strategy of the moose, ruminant physiology and the categorization of food quality.

## Introduction

Animals belonging to a variety of taxonomic groups, from slime moulds to gorillas, are now known to select the foods they eat to achieve a particular target nutrient balance [1–6]. They achieve this target balance by consuming a variety of foods and nutrients in specific relative quantities, and by compensatory feeding whenever this balance is not adequately approximated. By identifying an animal’s target balance, and the means by which it is achieved and defended, we can better predict foraging behavior [7], and thus improve the management of both wild and captive animal populations.

The moose (*Alces alces* L.) is an ungulate herbivore which ranges throughout the temperate and boreal forestlands of the northern hemisphere. Moose are classified as browsers, as defined by their distinct gut morphology [8, 9]. From early spring they carefully select leaves and forbs that are relatively easy to digest [10, 11], and then shift during the winter to a diet of twigs, needles and bark with lower nutrient content and digestibility [10, 12]. Both wild and captive moose prepare for this seasonal shift by reducing their metabolism and food intake during the fall, with associated adjustments to their rumen physiology [8, 13, 14]. A variety of nutritional influences on the diet choice of moose and other large herbivores have been suggested, ranging from the role of minerals [15], to plant secondary metabolites [16, 17] and macronutrients. For example, free-ranging moose, whose feeding time and energy expenditure are highly influenced by the environment, are suggested to select foods in a way that maximises their daily energy intake [18, 19]. The use of energy maximization as an assumption within optimal foraging models has been widely adopted, and has resulted in reasonably accurate predictions of foraging decisions by several ungulate species (e.g., [20–23, 24]).

Digestible energy is often the basis for such studies, and its use as a nutritional currency has produced a wealth of knowledge about herbivore foraging. However, counter-balancing the benefits of simplicity, this currency has the potential to hide underlying patterns attributable to the sources of calories in the diet. Although the main sources of energy for ruminant herbivores are digestible carbohydrates, including fermentable fibre [25], energy is also a property of the other macronutrients, both lipids and proteins. Therefore, when total digestible energy is used as a single currency, the functional roles and relative importance of the different macronutrients are left unresolved [26, 27]. Effective tools for modelling animal food selection in terms of these multiple currencies have only been developed relatively recently. The Geometric Framework for nutrition (GF) is one such tool [28]. The GF is an analytical framework that unifies several nutritional measures using simple geometric models to compare observed and predicted patterns of nutrient intake. It allows the researcher to study interactions between the different nutrients, and how the animal resolves the potential conflict between over-eating one or more macronutrients and under-eating others during periods of dietary imbalance (their “rule of compromise”, [28]). The GF is therefore well suited to testing the nutrient balancing hypothesis, which proposes that, when sufficient food is available, animal diet selection subserves the primary goal of obtaining a nutritionally balanced diet [28, 29]. The use of the GF in tests of the nutrient balancing hypothesis has provided unexpected insights for a variety of taxa, including herbivores, omnivores and predators [28], but has hitherto not been applied to the moose or any other ruminant.

**Fig 1:**
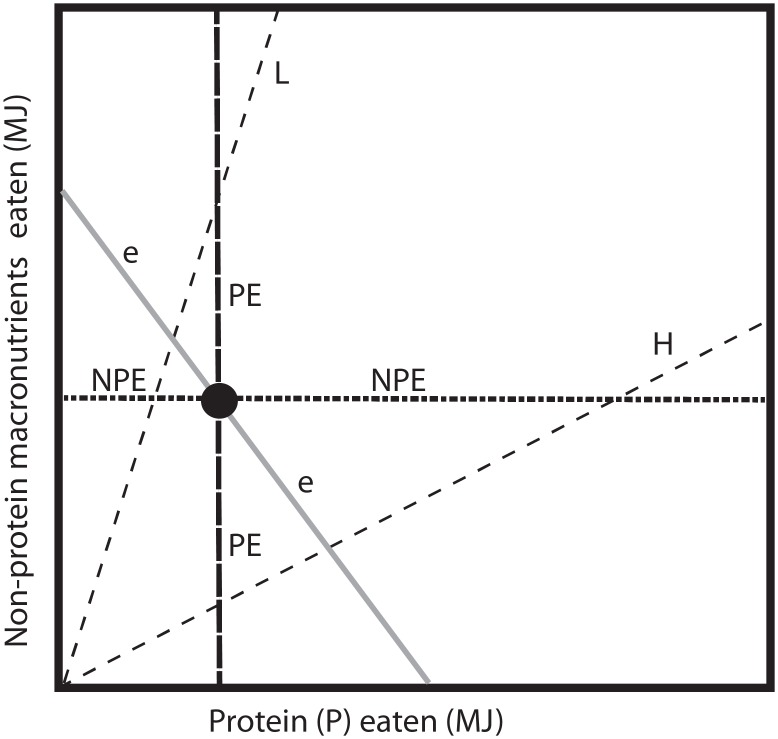
Logic of the experimental design under the Geometric Framework for nutrition (GF). The model shows potential outcomes when animals are fed diets containing different ratios of protein versus non-protein macronutrients (non-structural carbohydrates, lipids and fermentable fibers) [32]. Dashed radials (“food rails”) represent the nutritional balance of food items, here exemplifying two contrasting foods that are either relatively high in protein and low in non-protein macronutrients (H) or vice versa (L). When study subjects are given both of these complementary food items *ad libitum*, they are allowed to reach any point within the macronutrient space between the two food rails. Their intake during such a free-choice situation (“buffet”) indicates their preferred target point in macronutrient space (black dot) and provides a baseline for comparison with the rest of the experiment (the location of the target point in this figure is not empirically derived). If the moose’ intakes during buffets fall along line H, the strategy of protein maximization is indicated; if their intakes fall along line L it would indicate energy maximization; and if they combine the foods in specific non-random proportions the results would indicate macronutrient balancing (the black dot). Combined results from both buffets and periods when the moose are restricted to only one of the two food types allow us to see how the animals balance over-eating the nutrient in high concentration against under-eating the nutrient in low concentration, relative to their target, i.e. their rule of compromise. This pattern could potentially show regulation of total energy (*e*, grey isoline with slope of -1), or prioritization of protein energy (PE, black dashed vertical line), or prioritization of energy from non-protein macronutrients (NPE, black dotted horizontal line). (For a full description of the GF see [26, 28]).

Here we report a study that uses this approach to identify the nutritional factors that determine moose food choice during wintertime, in a situation without long-term deficiencies or limitations on foraging time. Even individuals that are not nutritionally deficient or time limited must choose what to eat and how much, and their choices are likely governed by the same evolved regulatory mechanisms (physiological feed backs) as their wild conspecifics [28, 30, 31]. In this study we manipulate the macronutrient composition of the diets of captive moose in order to test the nutrient balancing hypothesis: do the moose regulate their food intake towards a particular balance or do they instead try to maximize the intake rate of any single nutritional constituent (Fig 1)? The second aim was to measure the browsing response of moose when diets are imbalanced, to see if compensatory feeding takes place. The focus of our data interpretation is not on the absolute intakes by the moose, as these will likely differ between captive and wild settings. Instead we focus on the underlying patterns, because by identifying them, we are in a better position to understand the frames within which foraging choices of wild moose are made.

In the difficult trade-off between sample size, level of detail in observations and the associated financial costs, we decided to obtain high resolution data from a few individuals, similar to Belovsky’s pioneering work on moose foraging strategy [18, 19]. Unlike Belovsky, however, we followed recent studies [5, 33–35] in using continuous observations of focal subjects rather than assembling different model parameters from different groups of animals; for example, measuring intake rates in some and food choice in others. We provided the moose with unlimited amounts of foods that varied in their macronutrient content, while controlling for other factors that are known to influence their food choice in the wild, such as food plant density and distribution [36–39], mineral content [15], plant secondary metabolites [16, 17], and factors regulating bite size [22, 40, 41].

Using this approach, we predicted that moose that are allowed to mix their intake from two types of pellets varying widely in the ratios of protein to non-protein energy (non-protein macronutrients include carbohydrates and lipids; while lipids were held constant, carbohydrate content varied between pellet types), should choose the food with most non-protein energy if their aim is to maximize energy intake, as ruminants can most efficiently utilize energy from carbohydrates [25]. In contrast, the animals would choose the most protein rich food if maximizing protein intake. Alternatively, the moose will combine the foods in specific, non-random proportions if balancing their macronutrient intake, as would be consistent with the nutrient balancing hypothesis [28] (Fig 1). We also predicted that moose would alter their intake of browse, a minor but accessible food item, when only one of the two pellet types were provided, in an attempt to compensate for any resultant imbalanced nutritional state during these periods. Finally, we predicted that the food intake of the moose during such periods of restriction to a single pellet type would give us a preliminary indication of their “rule of compromise” (Fig 1). The aims of the study were achieved, and we discuss our results in the context of herbivore foraging theory and ruminant physiology.

## Materials and Methods

### Location, housing and subjects

The study was carried out between the 8^th^ Jan– 13^th^ Feb 2012 at the Skåne Zoo in southern Sweden (55°58’06” N; 13°31’52” E). The climate in this region is temperate with average temperatures during January and February ranging between -3 and 2°C [42]. Seven moose were allocated to two treatment groups (enclosure sizes 3.5 ha and 4 ha, S1 File). Group 1 consisted of three individuals and group 2 of four individuals (Table 1). Because the female yearling in group 2 was on several occasions observed to be scared off from food stations by other individuals, we could not assume that her consumption patterns were freely chosen and we thus excluded her data from the analyses. Adult females were both pregnant with twins. At the zoo the moose were normally given some newly harvested branches of broadleaved trees and both special game pellets (Renfor, ca 90% of mass given) and lucern pellets (alfalfa, *Medicago sativa*, ca 10% of mass given; 5–7 kg pellets in total/individual/24 h). Renfor is a game pellet often used to feed captive moose in Sweden.

**Table 1:** The age and weight of the individual moose at the Skåne Zoo involved in this study during January and February 2012, and the observed mean and maximum daily intake of pellets and browse across the whole experiment (variation among days indicated by ±1 SE).

Variables	Bull G1	Bull G2	Cow G1	Cow G2	Calf G1	Calf G2	Female G2 ^b^
Age	4 y, 8 m	1 yr, 8 m	3 y, 8 m	3 y, 8 m	8 m	8 m	1 y, 8 m
Weight (kg)	455	320	360	360	140	140	280
Date weighed ^a^	Nov-11	Oct-11	Nov-11			Nov-11	
Mean pellet intake (kg ww/day)	8.3 ± 0.44	7.3 ± 0.40	7.5 ± 0.39	7.0 ± 0.40	4.4 ± 0.25	5.2 ± 0.19	
Mean browse intake (g ww/day)	164 ± 19	188 ± 17	236 ± 20	172 ± 14	229 ± 20	225 ± 23	
Max food intake (kg ww/day)	13.2 ± 0.87	11.8 ± 0.59	11.7 ± 0.62	12.0 ± 0.64	7.3 ± 0.58	7.0 ± 0.23	
Mean pellet intake (g dm / W^0.75^/day)	76 ± 3.9	90 ± 3.8	81 ± 4.3	77 ± 4.2	97 ± 5.2	115 ± 4.4	
Mean browse intake (g dm / W^0.75^/day)	0.8 ± 0.09	1.2 ± 0.11	1.4 ± 0.12	1.0 ± 0.08	2.7 ± 0.24	2.7 ± 0.28	

G1 and G2 indicate which of the two groups (enclosures) the individuals belonged to.

^a^ Dates when four individuals were sedated and weighed. Weights for the other individuals were estimated.

^b^ female yearling not included in analysis due to anomalous behavior.

Bull G2 was a young adult and sexually mature (father of calves carried by Cow G2) and is treated as an adult male in statistical analyses. For definitions of maximum daily food intake, see Methods. “Day” equals 24 hours; W^0.75^ = metabolic body weight (kg); ww = wet weight; dm = dry matter.

### Ethical considerations

The study was granted an ethical permit by the Swedish Board of Agriculture (permit no. M 247–11). All sampling procedures and experimental manipulations were specifically approved as part of obtaining this permit. The owner of the land, the Skåne Zoo, gave permission to conduct the study on this site.

### Feed used in the experiment

Two types of pellets were specially produced for this experiment (by Lantmännen Lantbruk, Åhus) by modifying the proportions of the ingredients used in Renfor (Table A in S1 File). One of the pellet types had a higher concentration of carbohydrates (providing readily available energy, [25]) but lower concentration of protein (Low-protein, or L) than Renfor, whereas the other pellet type was relatively rich in protein and relatively poor in carbohydrates (High-protein, or H; Table 2). Concentrations of lipids, fibre, minerals and vitamins were kept at a similar level in both pellets (Table 2). We chose concentrations of carbohydrates and protein to create a wide range of possible nutrient blends in nutritional space (Fig 1) while not jeopardising the health of the moose (protein concentrations were always within the recommended range for moose, [43]). To avoid potential problems experienced by ruminants due to abrupt dietary shifts, the pre-procedural care consisted of a slow adjustment (four weeks) from limited food rations to food (their normal pellets) being available *ad libitum*. This was achieved without any health complications. The reverse was done post-procedure. In addition, ten days before the first buffet (the first week of the actual experiment), we gradually introduced the two new pellet types in limited amounts, alongside their regular pellets. At no stage during the experiment were any restrictions made in the total energy, essential nutrients or vitamins available to the animals. The variation in nutritional composition of the two diets was well within biologically suitable compositions for moose, and the exact diet composition and treatment duration was established in conjunction with veterinarians.

**Table 2:** Nutritional composition of pellet types and browse items from three *Salix* species eaten by captive moose at Skåne Zoo during the experiment.

	Pellets			*Fine twigs*			*Coarse twigs*			*Bark*		
	High protein	Low protein	Renfor ^a^	*S*. *ca*	*S*. *ci*	*S*. *f*	*S*. *ca*	*S*. *ci*	*S*. *f*	*S*. *ca*	*S*. *ci*	*S*. *f*
Nb pooled samples	4	4	2	8	1	4	1	1	4	3	1	1
Percentage dry mass	88.0	88.2	86.1	47.2	48.1	48.9	48.6	46.8	49.9	48.1	48.8	51.8
Ash	7.1	7.2	6.5	3.6	4.1	4.8	3.2	3.3	3.9	5.5	5.3	6.7
Crude protein	24.3	9.2	13.2	9.7	10.0	9.5	7.4	7.9	6.6	6.6	6.9	7.0
Available protein, AP	23.3	8.8	12.1	7.7	6.8	7.2	5.8	6.3	4.9	5.1	5.3	4.9
Ash-free neutral detergent fibre, aNDFom	32.1	32.5	32.3	54.1	52.3	51.7	56.4	57.5	58.3	40.6	37.9	35.5
Digestible neutral detergent fiber, dNDF (% of aNDFom)	51.5	57.0	54.2	28.5	26.3	32.7	26.0	25.2	27.4	45.5	51.7	57.4
Acid detergent fibre, ADF	13.4	13.6	12.8	48.3	48.4	45.3	50.7	51.7	50.3	37.0	34	28.9
Acid detergent insoluble N (% of totN)	3.9	4.4	8.8	26.7	33.4	24.7	27.2	34.4	24.2	26.3	18.5	11.2
Lipids	3.0	2.7	3.2	1.3	2.0	1.7	1.2	1.7	1.3	2.2	2.6	0.9
Tot non-structural carbohydrates by assay, TNC1	18.0	28.5	30.9	5.8	4.9	6.7	5.6	4.3	6.3	7.9	7.2	9.2
Tot non-structural carbohydrates by subtraction, TNC2 ^b^	33.5	47.8	44.7	30.7	32.4	32.6	31.1	28.7	29.4	44.7	47.2	50.4
**Protein energy, PE (MJ/kg)**	**3.9**	**1.5**	**2.0**	**1.3**	**1.1**	**1.2**	**1.0**	**1.1**	**0.8**	**0.8**	**0.9**	**0.8**
**Non-protein energy, NPE** ^b^ **(MJ/kg)**	**9.6**	**12.2**	**10.4**									

All nutritional variables are expressed as % of total dry mass unless indicated in brackets. *S*. *ca* = *Salix caprea*; *S*. *ci* = *S*. *cinerea*; *S*. *f* = *S*. *fragilis*; Number of pooled samples = for pellets the number represents weekly samples of the product; for each tree species material of fine twigs was pooled before chemical analysis according to harvesting date; coarse twigs either all together (*S*. *caprea*, *S*. *cinerea*) or by harvesting date (*S*. *fragilis*) depending on amount of material available; bark according to harvesting location.

^a^ The pellet Renfor included here for comparison;

^b^ TNC2 values for all 9 browse items likely overestimated and not used in calculations of NPE (see Methods).

To assess compensatory feeding, i.e. how the moose’ browsing behavior was influenced by the nutritional balance of their main diet, during all phases of the experiment each moose group was given eight freshly harvested tree branches (average weight 3 kg/ branch) in the morning (ca 8 am) and another eight branches in the afternoon (ca 4 pm). The trees were all *Salix*, a genus reported to be preferentially consumed by wild moose [10, 11, 37], but the particular species given depended on availability. The use of *Salix* was not based on any *a priori* knowledge of their nutritional content. The branches were harvested from dormant trees in the nearby region. Of the total number of batches 60% were *Salix caprea* (3 harvest locations), 30% *S*. *fragilis* (1 location) and 10% *S*. *cinerea* (1 location).

### Experimental design

The experiment was designed around the application of the Geometric Framework for nutrition [28]. We identified the moose’s self-selected target in nutrient space by allowing them to compose a diet by mixing two complementary pellet types (hereafter called a “buffet diet”, Fig 1). Such periods of free choice were intermixed with periods when they were restricted to one of the two imbalanced pellet types (“no-choice diet”), to allow for an assessment of compensatory feeding and a preliminary identification of their rule of compromise. The experiment consisted of five consecutive phases (each 7 days): i) first buffet diet; ii) no-choice diet (either H or L); iii) buffet diet (Post L buffet or Post H buffet); iv) no-choice diet (either H or L); v) buffet diet (Post L buffet or Post H buffet). We expected that homeostatic responses based on physiological states would be expressed within periods considerably shorter than 7 days [7], as this has been shown to be the case in several mammal species [1, 34, 44, 45]. During all phases pellets were given *ad libitum* and browse in limited amounts, as described above. Both groups of moose shifted diets at the same time, but when group 1 was given L, group 2 was given H and vice versa. By alternating weeks of buffets with weeks of no-choice diets, potential post-restriction compensation responses may be revealed. The first buffet (Buffet 1) was used as the reference value as the moose had not yet been put through any restricted diet that could affect their voluntary intake. We define their intake target as the moose’ mean intake across all days of Buffet 1, to acknowledge that post-ingestive feedbacks can operate on a scale longer than 24 hours [30].

### Measurements of food intake, feed rates and browse availability

In each enclosure moose were given pellets in buckets through openings in the walls of a small shed. The feeding quarters of the two groups were out of sight of each other. There were surplus buckets available, thus always more than enough feeding places present for all individuals at all times. During buffet phases extra buckets were used (the number of buckets per pellet type therefore remained the same), and we changed the specific placement of the different pellet types daily. To measure food intake we weighed the content of each bucket using a hanging scale (Kern accuracy 10g) before and after each re-filling. To obtain individual feed rates (g eaten/min), we also weighed the bucket contents after individual feeding sessions during daytime (tot 498 such feed rates were collected). For each week-long experimental phase (first buffet, low-protein diet, post-L buffet etc.), an average feed rate was calculated for each individual moose and pellet type.

To observe the moose’ browsing response we placed branches horizontally upon iron structures 2 m above the ground and accessible by all individuals. To obtain detailed rates of browse intake, we observed the moose opportunistically during each experimental phase while they were consuming items from the branches provided. Close-up focal scans lasting 1–3 minutes provided data on the number of bites/min and the length (cm) of twig and bark strip/bite (tot 428 such feed rates were collected). Averages were calculated for each moose age/sex class, browse species and item category.

To estimate daily browse availability we weighed each batch of branches before being given to the moose (Ohaus Defender 3000 floor scale, accuracy 10g). We used data from representative sample branches for each batch to calculate the average g of edible twigs/kg branch (wet weight) of the three different *Salix* species, using maximum twig diameter limits as defined below (as per [46]). This was multiplied with the total weight of batches to obtain an estimate of how much twig material was available to the moose during each 24 hour period. To estimate browse utilization, one browsed branch per batch was specially studied with respect to the proportion of twigs that had been eaten and the maximum diameter of bite surfaces (to the mm, using a caliper).

### Potential constraining factors; gut capacity and air temperature

To estimate whether individuals fed to maximum gut capacity during the experiment, a potentially constraining factor on their diet choice, we calculated the mean daily intake of the days with the top 10% of total food intake. These values were then qualitatively compared to published data on the normal upper range of daily wet weight food intake by adult moose during winter time, which varies between 12 and 21 kg (reviewed by [47]). To test whether temperature influenced observed feeding choices we obtained air temperature data (C° recorded every 3 hours) from a weather station run by the Swedish Meteorological Service located nearby (Hörby). Based on those data we calculated the mean air temperature for each 24 hour period in the experiment.

### Chemical analyses

For nutritional analyses we collected plant samples that were consistent with the item type and bite sizes observed to be eaten. In conjunction with supplying browse each morning and afternoon, a ninth branch was randomly selected as an unbrowsed representative of each batch. From these branches, twigs and bark strips were measured and weighed before storage in a freezer (S1 File). Pellet, twig and bark samples were prepared and analysed at the Kungsängen Research Centre (the Swedish University of Agricultural Sciences, Uppsala, Sweden). Because the diameter of twigs greatly influences the degree of digestibility [48, 49], we divided the defrosted twigs into two categories: “fine” (< ca 3 mm diam) and “coarse” (> ca 3 mm; S1 File). Length of twigs and bark samples was measured and wet weight recorded before drying at 60°C for 48 h. After 24 h in room temperature they were weighed again. Pellet samples were dried and weighed using the same approach. The dried material was ground using a hammer mill (KAMAS Slagy 200B; 1 mm sieve) and samples were pooled (Table 2).

To calculate the total available protein and non-protein energy, we measured nitrogen, lipids, ash (total minerals), digestible carbohydrates and non-digestible fiber (assayed both as acid-detergent fiber and ash-free neutral detergent fiber, see below). We analysed concentrations of ash, total nitrogen, crude fat (hereafter called “lipids”) and acid-detergent fiber (ADF) using conventional laboratory techniques (S1 File). To estimate the portion of non-digestible protein, we measured insoluble nitrogen remaining in the acid-detergent fiber fraction using the Kjeldahl method [50]. Available protein (AP) was calculated as total protein (total nitrogen multiplied by 6.25) minus non-digestible protein present in acid-detergent fiber [51]. Digestible carbohydrates (sugars, starches and digestible fiber) were analysed using three different methods. Water soluble carbohydrates and starch were determined enzymatically [52]. We estimated total non-structural carbohydrates (TNC) both by assay (TNC1 = water soluble carbohydrates + starch) and by subtraction (TNC2 = (100—(NDF+AP +lipids+ash)) [53]. We judge TNC1 to be the most reliable alternative with regards to the browse items in this study, as their unexplained fraction was very large (Table 2) and likely represented pectin, organic acids and unidentified fats instead of non-structural carbohydrates. TNC by subtraction does not account for the presence of vitamins, plant secondary metabolites and other minor amounts of non-carbohydrate substances [53]. However, in the case of the pellets used in this study we judge TNC2 as being the most reliable estimate on which to base energy calculations, for three reasons: 1) the above mentioned plant fractions are likely small in this type of material (for vitamins see Table A in S1 File), 2) the unexplained fraction of dry matter was relatively small, and 3) the TNC2 values closely resemble the equivalent fraction declared by the manufacturer (Table A in S1 File). We present both values in Table 2, but use TNC1 in data analyses including browse, and TNC2 in analyses including only pellets.

Samples were analysed for ash-free neutral detergent fibre (aNDFom) using heat stable α-amylase and sodium sulphite, and crucibles instead of filter bags [54]. To estimate in vitro digestibility of aNDFom, dried feed samples were incubated for 96 h in buffered moose rumen fluid from wild moose (described in detail in [55]) using an automated gas-*in vitro* system [56]. All samples were incubated in three replicates within one run. After the incubation all residues were analysed for aNDFom to identify the proportion of total aNDFom that was fermentable for the moose (dNDF). This was done at the Dept. for Agricultural Research for Northern Sweden (Swedish University of Agricultural Sciences, Umeå).

### Video surveillance and analysis: feeding times and browse bites

Food intake was monitored 24 hours a day, by placing surveillance cameras (Zavio D7110, 5 in total) above each cluster of feeding places of pellets and browse. The cameras had motion detectors and were triggered by movement to record film, day and night (IR LED lights). For the five week duration of the experiment, 32 days of continuous 24-hour camera observation were recorded.

All filmed feeding sessions were analysed using Zavio software by AMF. The start- and end time of each individual’s pellet feeding session at each bucket (H or L) was noted to the second. The watch was stopped at any break in eating longer than 30 seconds. With respect to browse, the start- and end time of each individual’s feeding session was noted to the minute. The number of bites was counted and categorised into three classes: fine twig, coarse twig and bark strip. The distinction between fine and coarse twigs was made visually, guided by our measurements (see above). The bark bites were registered as being either of average length (15–20 cm, as per live observations) or as “gnaws” (1–2 cm long attempts to gain hold of a bark strip).

### Additional data analysis

For those feeding sessions for which we did not have weight measurements of buckets before and after feeding by separate individuals (see description of feed rates above), we calculated the amount of pellets consumed by each individual per session by multiplying the duration of the feeding session with the matching feed rate. The weight of browse ingested by each individual per session was calculated by multiplying the number of bites with average bite sizes and data from collected plant samples (g/cm, wet and dry weight). To determine whether the moose’ self-selected intakes were underpinned by random or non-random feeding, a fundamental step in the identification of their nutritional strategy, we assessed the intake data using meals as the unit. Separate feeding events that occurred with a gap of 30 minutes or less of each other were defined as belonging to the same meal. We calculated the number of meals per 24 h and the total amount of food ingested per 24 h. We calculated the nutrients ingested by each individual during each meal by multiplying the total dry matter (dm) ingested of each food category (pellet type H and L, fine twig, coarse twig and bark) with the matching nutritional fraction (g per 100g dm).

For the sake of uniformity in the geometric analysis of the moose nutrient intake, we use energy units (MJ) for all macronutrients, although mass units could equally have been used. We calculated the energetic value of each food using the following conversion factors [57]: 37.7 kJ/g lipid and 16.7 kJ/g available protein (AP) and digestible carbohydrate (i.e. total non-structural carbohydrates (TNC) and digestible fiber (dNDF)). With regards to pellet intake, protein energy (PE) is defined as the energetic value of available protein; and non-protein energy (NPE) as the energy sourced from TNC2 + lipids + dNDF. We do not use the unit “digestible energy” as this does not distinguish the macronutrients and therefore obscures any potential interaction between them.

To check whether the moose reached a stable nutritional intake within the duration of an experimental phase (7 days), we plotted the mean intake ratio PE:NPE per 4 hour interval across the whole first buffet. To relate the moose’ nutritional target to the composition of the pellets and browse items used in this study, we plot AP and non-protein macronutrients (TNC1 + lipids + dNDF) in terms of %dm, using food rails and target points [28] (Fig 1). We analysed the moose’ rule of compromise by using our estimates of PE and NPE ingested (pellets only) per individual per kg metabolic body weight (W^0.75^, [58]).

### Statistical analysis

Because we were limited to a few captive moose for this experiment, we maximised statistical power using a mixed model design in which the factor of primary interest (dietary treatment) was a within-subjects effect and the controlling factor (order of presentation of the diets to the two groups) was the between-subjects effect. In this design, non-significant outcomes would be difficult to interpret with any certainty because of limited statistical power, but positive outcomes (e.g. observed dietary selection vs. random) would provide support for nutrient-specific selection in moose.

We calculated the cumulative PE:NPE of pellet meals ingested by each individual across the seven days during the first buffet phase (as per [34]). To test whether the moose’ self-selected intakes indicated random or non-random feeding, we compared PE:NPE of these cumulative meals (the number of data points = the total number of meals during the week per individual minus 1) with ratios that represented the same meal volumes (tot dm ingested) but equal amounts of each of the two pellet types. This is referred to as the “equal intake scenario”. We used a Generalized Linear Mixed Model (GLMM) with this equal intake scenario as an offset variable implying that the intercept-only model tests if the ratio between PE and NPE significantly differs from this scenario. The GLMM was fitted with a Gamma distribution and log-link.

To test whether the moose’ intake of pellets (tot g dm/individual/ W^0.75^/day) was influenced by treatment phase or mean daily temperature, we analysed the data using Linear Mixed Models (LMMs). Similarly we used LMM to assess whether their total intake of browse (tot g dm/individual/ W^0.75^/day) was influenced by treatment phase, mean daily temperature, or tree species offered (analysis Browse A). The model with the lowest Akaike Information Criterion (AIC; [59]) value was chosen as the best model. For both pellet and browse intake (g dm/individual/ W^0.75^/day) we performed post-hoc tests to check for differences between the treatment phases using the “planned contrast” approach with FDR correction [60]. We also used LMM to test whether the pattern of browse intake could be explained by the variation in daily availability of edible twigs (“browse availability”; analysis Browse B). This variable was not included in the model described above as the sample size was different (45 of the 186 24h periods lacked matching browse availability data). To assess how daily intakes of PE and NPE (MJ/ W^0.75^/day) by adult individuals differed between the treatments we also used a LMM, followed by a “planned contrast” post-hoc test to specifically identify how intakes differed among the three free-choice phases (buffets), and the two no-choice phases of the experiment. Enclosure and individual (adults only) were used as random variables in both the GLMM and LMMs and all statistical analyzes were performed in R 3.0.1 [61]. For details, see S1 File. Protein and mineral concentrations in plants can be correlated [5], making it difficult to separate these two constituents as driving factors behind food choice. We therefore conducted Pearson correlation tests between the variables ash and AP (% dm, both variables log transformed).

## Results

The moose in our study ingested on average 5–8 kg ww/day (Table 1). Pellets represented 98% of moose food intake by weight, as individuals of all ages ingested an average of only 202 g ww (± 8.0 g) of browse per day (40–50% of estimated available edible browse depending on the group, averaged across the study). Counting only the minutes of actual food ingestion (excluding breaks > 30 seconds; cut-off used to enable matching with feed rates), the moose spent on average 57 minutes/24h ingesting pellets, divided among 4–11 separate meals (mean 6 meals/24 h). The time individuals spent eating browse ranged between 13–205 min/24h. Each group’s individuals normally fed together. The nutritional composition of the food types used in this experiment is presented in Table 2. Ash was not significantly correlated with AP in the food items in this study (Pearson correlation, pellets included: p = 0.232; only browse: p = 0.183).

**Fig 2:**
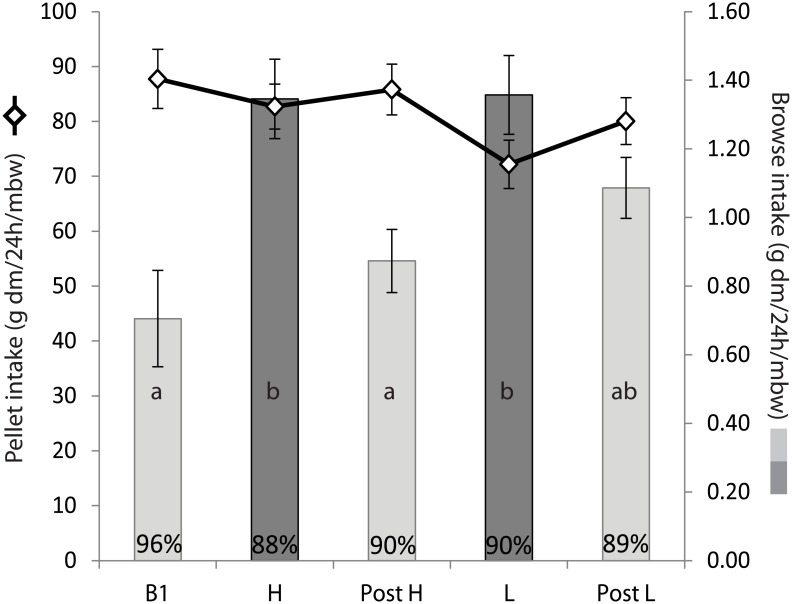
Mean intake of pellets (diamonds, y_1_) and browse (bars, y_2_) by adult moose; for each of the five experimental phases (high protein (H), low protein (L), Buffet 1, Post H buffet, Post L buffet). Variation among days and individuals is indicated by ± 1 SE. Intake of browse during no-choice treatments (H and L) is indicated with dark grey bars and compared to browse intake during buffets (light grey bars); statistical results regarding browse are indicated by letters within bars (see also Table 4). Values at bottom of bars show the percentage of the browse intake that was made up by fine twigs, the remainder being coarse twigs and bark.

The observed maximum daily intakes by individuals (mean of the top 10% of daily observations, Table 1) was within the normal range of daily food intake by adult moose during winter time (up to 21 kg ww reported, reviewed by [47]). The model selection based on lowest AIC shows that air temperature did not explain the moose’ intake of total pellet mass, but that treatment as the lone variable provided the best model (Table 3, model parameters in Table A in S2 File). The post-hoc test showed that there were no significant differences between treatments in total intake of pellets across the whole experiment (Table 4, Fig 2).

**Table 3:** Alternative linear mixed models (LMM) that test which variables influenced the moose’ intake of pellets and browse.

Model	Variable 1	Variable 2	Variable 3	AIC
Pellet intake (186 obs)				
LMM 1	Treatment	Temperature		86.8
*LMM 2*	*Treatment*			*77*.*0*
Browse intake A (186 obs)				
LMM 1	Treatment	Temperature	Tree species	308.3
LMM 2	Treatment	Tree species		301.9
LMM 3	Treatment	Temperature		302.4
*LMM 4*	*Treatment*			*294*.*7*
Browse intake B (141 obs)				
*LMM 1*	*Browse avail*.			*254*.*4*

The LMMs test whether the intake of pellets and browse (g dm/individual/ W^0.75^/day; tot 186 24h-periods) was influenced by treatment (the five different week-long dietary regimes tested), mean daily temperature or tree species (three species of *Salix*). A separate LMM was performed for browse intake with 141 matching observations of browse availability (g edible browse/batch). Within group, the model with lowest AIC was chosen as best model (italics) for which model statistics are shown in Table A in S2 File. AIC values cannot be compared among the three groups of models.

**Table 4:** Post-hoc test results of the best linear mixed models regarding the relationship between daily pellet and browse intake and dietary treatment.

Comparisons	Pellet intake LMM2		Browse intake A LMM4	
	z-value	p-value	z-value	p-value
H—L	-2.018	0.257	-0.269	0.788
H—Post H buffet	0.348	0.997	2.642	0.016 *
Buffet 1 –H	0.637	0.969	-3.949	<0.001 ***
L—Post L buffet	1.553	0.527	1.755	0.119
Buffet 1 –L	2.520	0.086	-4.200	<0.001 ***
Post H—Post L	-0.814	0.926	-1.156	0.297

Post-hoc test results show the relationship between daily pellet intake and dietary treatment (in Table 3, and Table A in S2 File, this model is called Pellet intake LMM2), and daily browse intake and dietary treatment (called Browse intake A LMM4 in Table 3, and Table A in S2 File). The post-hoc tests compares pellet and browse intake (g dm/individual/ W^0.75^/day) during the five different treatments (high protein (H), low protein (L), Buffet 1, Post H buffet, Post L buffet).

*p<0.05;

***p<0.001.

### Nutritional intake by moose

The moose did not select randomly between the pellet types. The intake ratios of accumulated meals by individuals during the first buffet (relevant for identifying their nutritional intake target) differed significantly from the null model of equal intake (GLMM t-value -4,662, p < 0.001). The duration of the experimental phases (7 days) appeared to be sufficient in order to see homeostatic responses, as the moose intake ratio PE:NPE stabilised around 30–60 hours into the first buffet (Fig A in S3 File).

**Fig 3:**
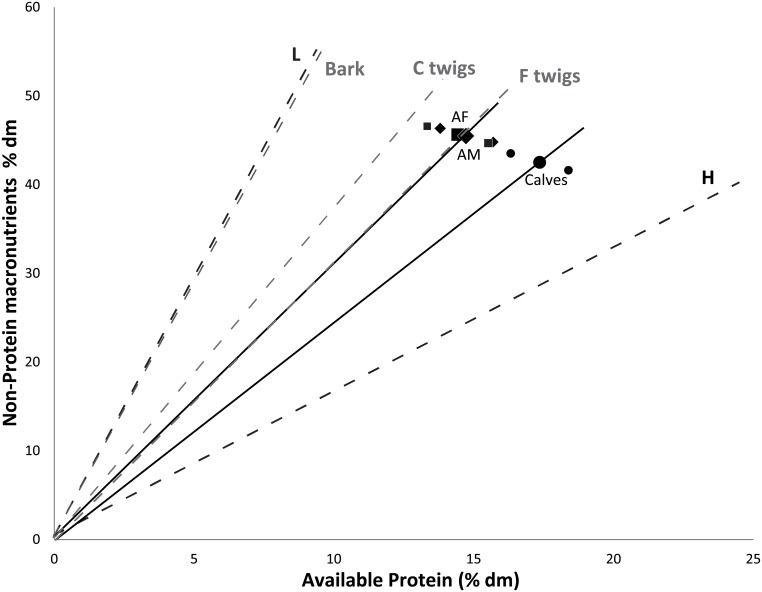
The nutritional target of captive moose in relation to food pellets and browse items. Pellets provided the moose with 98% of their total intake. The average balance between available protein (% of total dm ingested) and non-protein macronutrients (% of total dm ingested of TNC1+lipids+dNDF, see text for definitions) in the pelleted food ingested per 24h period by adult females (AF), adult males (AM) and calves during the first buffet week of the experiment indicate their preferred nutritional state (large solid square = mean for all AF, small solid squares = mean for each individual AF; large empty diamond = mean for all AM, small empty diamonds = mean for each individual AM; large solid dot = mean for all calves, small solid dots = mean for each individual calf). The mean trajectories of adults and calves are indicated with solid black lines. Dashed black food rails show the composition of the low-protein pellets (L) and high-protein pellets (H). Had individuals chosen to eat only one of L or H, their resulting point would have fallen somewhere along one of these two lines (Fig 1). Dashed grey food rails show the average composition of coarse twigs (C twig), fine twigs (F twig) and bark from three *Salix* species (Table 2).

The moose did not consistently choose the food containing the most readily available energy (digestible carbohydrates), or the food containing the most protein (Fig 2). Instead they combined foods in specific proportions to provide a particular ratio and amount of macronutrients. The mean macronutritional ratio between protein intake (% available protein (AP) of total dm) and non-protein intake (% TNC1 + % lipids + % dNDF of total dm) by adult individuals during the first buffet was 0.30 (14:46) for females (cow 1 = 0.29, cow 2 = 0.35) and 0.31 (14:45) for males (bull 1 = 0.35, bull 2 = 0.30). The calves ingested a higher proportion of protein than adults (mean 0.40 (17:43), 0.45 and 0.38 respectively), although statistical significance was not tested due to sample size. Assuming that this was the result of unconstrained *ad libitum* dietary selection, we defined this as their intake target. Expressed in terms of energy (PE:NPE), the intake target was 0.22 (16:73) for females, 0.22 (20:93) for males, and 0.28 (11:40) for calves. The moose’ intake target, in particular the target of the adults, was similar to the composition of fine *Salix* twigs (Fig 3). In other words, by combining the H and the L pellets the way they did during the buffet, they achieved a similar nutritional balance as they would have achieved by eating only these twigs.

**Fig 4:**
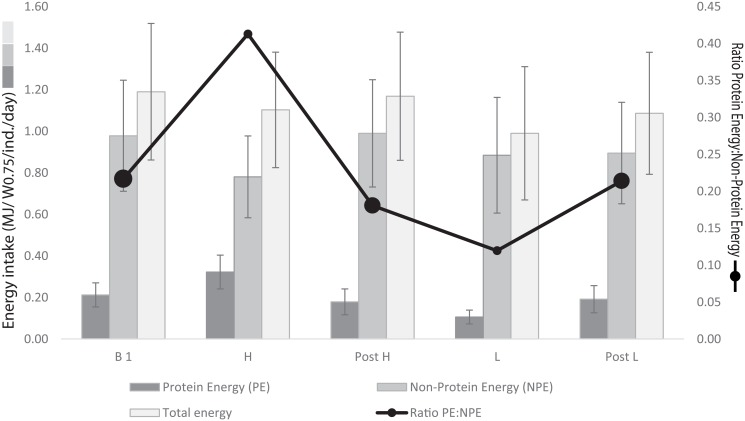
Mean daily intake of protein energy (PE, dark bars, y_1_), non-protein energy (NPE, medium dark bars, y_1_) and total energy (light bars, y_1_) by adult moose (±1 stand. dev) during each of the five experimental phases (high protein (H), low protein (L), Buffet 1, Post H buffet, Post L buffet). The moose’ daily intake ratio PE:NPE is shown on the y_2_ axis (black dots and lines), with intake during buffet phases indicated by extra-large dots. The moose maintained a similar PE:NPE ratio during each of the three buffet weeks (statistical results in Table 5).

**Fig 5:**
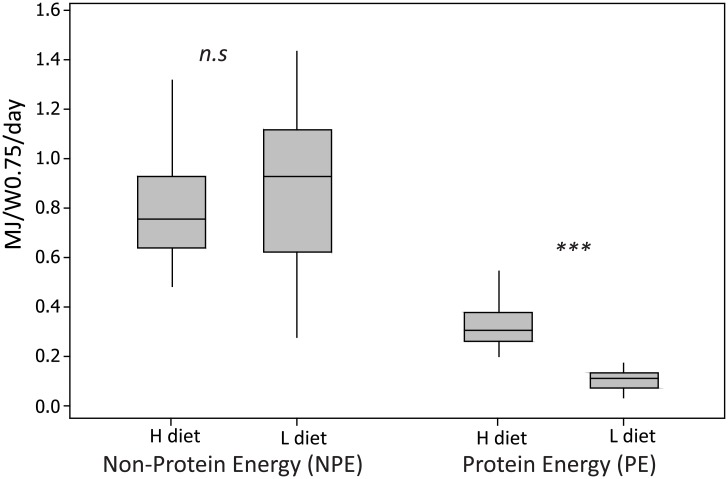
Interquartile box plot of the daily macronutrient intake by moose during the two no-choice phases of the experiment (the box represents the middle 50% of the data, whiskers represent the upper and lower 25% of the distribution). Daily protein energy (PE) intake per adult moose (MJ/W^0.75^/day) differed significantly between the two no-choice phases of the experiment (H diet: high protein, low carbohydrate; L diet: low protein, high carbohydrate), but the intake of non-protein (NPE) did not (statistical results in Table 5). Non-protein macronutrients include TNC1, lipids and dNDF (see text for definitions).

Averaged across all individuals and treatments, the non-protein consumed (dm) was to 41% made up by dNDF (± 0.2%), 53% by TNC1 (± 0.3%), and 6.4% by lipids (± 0.1%). The daily intake ratio PE:NPE by moose across all days during the entire experiment spanned the range 0.12–0.41 (Fig 4). The moose maintained a similar PE:NPE ratio during each of the three buffet weeks (Fig 4, Table 5). Daily PE intake per adult individual (MJ/ W^0.75^/day) differed significantly between H and L treatments, but the intake of non-protein did not (Fig 5, Table 5). In other words, intake by the four adult moose resemble the prediction of non-protein energy prioritisation, as they under-ate or over-ate protein compared to their target. Based on the results from this experiment we cannot conclude that the moose show post-restriction compensation, i.e. that they modify their pellet choice in the week following a dietary phase during which they were restricted to a single pellet type (Post L and Post H) according to any consistent pattern.

**Table 5:** Post-hoc test results of two linear mixed models (LMM) regarding adult moose individuals’ daily protein energy intake (PE) and non-protein energy intake (NPE) in the experiment (125 observation in each model).

Comparisons	Protein energy (PE)		Non-protein energy (NPE)	
	z-value	p-value	z-value	p-value
H—L	12.146	<0.001***	-1.560	0.356
Buffet 1 –H	-4.499	<0.001***	2.355	0.111
Buffet 1 –L	6.988	<0.001***	0.880	0.533
Buffet 1 –Post H buffet	1.715	0.130	-0.437	0.662
Buffet 1 –Post L buffet	1.206	0.273	0.766	0.533
Post H buffet—Post L buffet	-0.539	0.590	1.272	0.407

For model parameters of the LMM see Table B in S2 File. The post-hoc tests compares PE and NPE intake (MJ/individual/ W^0.75^/day) between the two no-choice phases of the experiment (high protein (H) and low protein (L)), between these two phases and the first buffet (Buffet 1), and among all the three buffet phases (also Post H buffet, Post L buffet).

*p<0.05;

***p<0.001

### Selection of browse

Individuals’ pattern of browse intake could not be explained by variations in mean daily temperature or the species of tree offered (Table 3). Neither could it be explained by the amount of edible twig available (LMM t-value 0.846, p = 0.400, Table A in S2 File). Treatment was however a significant variable in the best model (Table 3), and their intake of browse was significantly higher during the no-choice phases than during buffet phases (Fig 2, Table 4). One exception was that browse intake by adults was not significantly different between L and the Post-L buffet. The intake of browse was dominated by fine twigs (88–96% of adults’ daily intake of browse, Fig 2).

## Discussion

Our experiment produced three key results. First, food intake by the captive moose when given free choice appeared consistent with reaching a particular balance between macronutrients, and thus lends further empirical support to the nutrient balancing hypothesis [28, 29]. Second, when restricted to a diet either too high or too low in protein relative to the self-selected diet composition under free-choice conditions, moose increased their intake of the food item closest in composition to the self-selected ratio available, in this case twigs of fresh *Salix* branches. Third, when the moose were restricted to a diet that differed in composition to the self-selected ratio under free choice conditions, they consumed pellets in a manner which maintained a relatively stable intake of non-protein macronutrients and in so doing had a wide range of protein intakes. These results have several potential implications for foraging theory and herbivore ecology.

### The nutritional strategy of moose

During buffet phases in our experiment, the moose had unlimited access to pellets with complementary nutritional qualities. These pellet types would both conventionally be classified as “high-quality” due to their being easily digestible, palatable and of suitable bite size. Individuals did not specifically select either the most carbohydrate-rich or the most protein-rich foods that were available to them (fat concentrations were held stable), but mixed their intakes during each meal. In other words, the moose did not attempt to maximize their intake of energy or protein under these experimental conditions. Their accumulated non-random intakes during the first buffet week, our reference point against which we compare the other treatments, suggest active regulation rather than switching between the two foods randomly. Regardless of their previous diet, when moose were returned to a free-choice situation they mixed foods during the week and achieved a consistently similar point in nutrient space (Fig 4), indicating that this is their preferred nutritional balance and absolute level of intake under the environmental circumstances at the time (the daily intake of available protein by moose in this study is in accordance with estimated requirements, [43]). Our study thus lends support to the nutrient balancing hypothesis. Similar target seeking feeding behavior has also been reported for several other species of mammal (e.g. [45, 62]). Although nutrient balancing has not previously been demonstrated using the Geometric Framework for Nutrition (GF) in any other species of large herbivore, there have been several observations which indicate how important diet mixing is for ungulates at various spatial and temporal scales (e.g., [29, 63, 64–66]).

During two weeks of the experiment the moose were restricted to a diet that had a protein:nonprotein ratio either too high or too low relative to their identified target. When we compare the resultant intake from these “no-choice” weeks with the “free-choice” weeks we can make a first attempt to identify their nutritional priorities under balance constraint, or rule of compromise [28], acknowledging that further studies with more individuals and a larger number of different diets are needed to confirm the results. In relation to the model predictions, the moose were highly flexible in their protein intake, while maintaining a relatively stable intake of non-protein energy (Figs 4 and 5, Table 5). In effect, it was the intake of total digestible carbohydrates that was maintained in this experiment, as fat was held constant in both treatments. The results thus indicate that the moose’ feeding priority was to defend the non-protein energy target (line NPE in Fig 1), rather than to prioritise total energy intake regardless of its nutritional substrate.

Relatedly, other experimental captive studies have found that moose and deer maintain a stable intake of (total) digestible energy despite of significant variation in food composition (moose, [67], fallow deer, [68], red deer, [69, 70]). In other words, the animals in these studies did not appear to maximize their energy intake but instead regulated their energy intake around some goal; but because all kinds of digestible energy were combined into a single currency, any specific macronutrient prioritization cannot be inferred. As we found in this study (Fig 4), total energy intake by individuals may be more or less constant across varying diets even when interesting differences in the nutritional balance are hidden beneath this composite value. Therefore, nutritional interactions, like the pattern of macronutrient prioritization which we have shown here, are only possible to identify by separating the macronutrients in the analysis. Another prerequisite is to conduct detailed observations of everything individual animals eat over long periods. This is of course easier to do using captive rather than free-ranging animals, even though the captive setting requires caveats. However, whereas captive conditions differ from the wild, the nutritional strategy observed in our study nevertheless likely reflects physiological processes within the animal that are associated with fitness benefits selected for over the species’ evolutionary history, and might be presumed to be phenotypically conserved [28, 30, 31]. Although there are few direct tests of this, the tight congruence between macronutrient ratios selected in captive experiments on domestic cats eating manufactured foods [44] with the diet of free-roaming feral cats [71] is strongly suggestive.

Our study had the potential to observe “post-restriction compensation”, in which compensatory feeding takes place after a period of imbalance [72]. The moose’ intake of pellets did not indicate such a response. The only tentative indication of such behavior was the sustained high intake of browse that occurred during the Post L buffet (Fig 2). However, this observation alone is insufficient to draw a clear conclusion and it remains possible that moose instead have physiological mechanisms for dealing with such enforced imbalances under the conditions assessed or that a treatment period longer than 7 days may be needed to see more definitive patterns of post-restriction compensation in a ruminant. A goal for the future is to expand studies like ours using longer time series and also sample sizes that provide more confidence in identifying the variation in nutritional responses among age-sex classes. Elucidating these patterns would also benefit from the use of additional dietary options providing more food rails within the Geometric Framework.

### Compensatory feeding of browse: implications for ranking food items

While the moose in our study ate similar amounts of pellets throughout all experimental phases, they significantly increased their intake of browse items during no-choice treatments (Fig 2). This pattern could not be explained by changes in air temperature or differences in *Salix* species offered. The pattern observed is unlikely to be predicted by conventional food quality classification, because relative to the pellets, the browse items were low in energy and high in fibre (and likely higher concentrations of plant secondary metabolites, [17]) and thus conventionally classified as so called “low quality” food items. Instead we suggest that the moose increased their intake of browse items during no-choice phases because the nutritional balance of these items overlapped with the moose’ nutritional goal (Fig 3). This observation is also consistent with moose regulating their nutrient intake. During no-choice treatments the twigs were the only means available for the moose to attempt to get closer to their self-selected target in nutritional space, and we argue that this is why they ate more twigs during these weeks than when they had access to a buffet. As the browse was provided in limited amounts it was likely not possible for individuals to actually reach this target point during no-choice phases. Even though the twigs were just as well balanced for the moose when they returned to a buffet, the need for such compensatory feeding was reduced due to the presence of nutritionally complementary pellet types, and they could again browse as they normally did. Food items which appear to be particularly well-aligned with an animal’s intake target balance, have been identified previously by geometric analysis, for example in studies of frugivorous primates [73] and birds [74]. Further research is needed to identify which other tree species, besides *Salix*, provide well-balanced items for moose, with the outcomes potentially relevant for our understanding of how moose utilize different tree species and the associated damage in, for example, production forest stands.

Hence, a food item that conventionally would be categorized as being of “low quality” (like the dormant woody twigs) may for some herbivores, sometimes, be of “high quality” due to the particular palette of nutrients and structures they contain (e.g. similar evidence from dairy cows, [75]). Instead of supporting such categorical classifications, our results support the idea of complementarity [30, 64, 76]. The value of a particular food is not necessarily an inherent property, but instead varies depending on the animal’s physiological state. This is because when an animal is regulating towards a balanced macronutrient intake, as mediated by interactions between the senses and the viscera [30], the limiting and thus desired component(s) can change rapidly [4]. Only foods that closely match the nutritional requirements of the animal can be argued to have a relatively stable value, making such foods more likely to become a staple in their diet [30, 77]. This idea of complementarity may explain the variation between studies concerning which food items are most preferred by moose [12, 37, 78], and their preferential selection of relatively protein-rich items in some cases [46, 79, 80], but not in others [16, 17, 37, 38].

### Physiological considerations

Our finding that the moose prioritized a stable intake of carbohydrates in the face of dietary imbalance may have its explanation in ruminant physiology. The lower threshold is thought to be set by the rumen microbes’ need for energy, which makes them quickly deplete the glucose providing carbohydrates ingested [25]. At the same time, ingestion of too much non-structural carbohydrates is problematic for the ruminant’s digestion, as rumen pH then declines, potentially causing ruminal acidosis [75]. The contrasting capacity of moose to vary relatively widely in their daily protein intake may be because they obtain most of their daily protein from the microbe biomass, rather than the food [25]. When nitrogen in the food is in short supply, rumen microbes can efficiently use ammonia, whereas during excess nitrogen supply, the additional nitrogen can be excreted as urea. The price for this flexibility is the energy required to regulate nitrogen levels [43, 81], which act to further enhance the host’s need to acquire sufficient glucose. This flexibility in protein intake does not however negate the potential for free-ranging moose populations in marginal habitat to be seasonally nitrogen deficient [82]. Below some minimal threshold of protein intake the compensatory capacity of the ruminant’s micro-organisms will not be sufficient; a threshold which our study subjects never approached.

Selection for a well-balanced food item (the *Salix* browse) during no-choice phases was likely also influenced by the need to ensure proper rumen function. Ingestion of either too much carbohydrates or too much protein leads to a shift in the pH of the rumen, which may lead to suboptimal digestion [75, 83, 84]. Dietary fibre appears to have contributed to balancing the macronutrient composition of the *Salix* twigs relative to the moose intake target, as its inclusion on the NPE-axis contributed to the overlap of the twigs with the intake target (Fig 3). Ruminants require a continuous intake of fibre to ensure the continuous existence of cellulolytic microorganisms in the rumen and to stimulate secretion of acid-neutralizing saliva [85]. As the acid production in the rumen is due primarily to fermentation of carbohydrates, the balance between digestible carbohydrates and structural fibre should be of great importance in this regard [75]; just like the balance between protein and carbohydrates is important for microbial nitrogen metabolism and for detoxification of PSMs [86]. Furthermore, although our study subjects did not eat large amounts of *Salix* twigs, when eaten in large amounts, tannins found in these twigs may have a direct positive effect on rumen macronutrient imbalance, as tannins can bind both to proteins and carbohydrates with high molecular weight [87], possibly alleviating dramatic shifts in rumen pH.

### Conclusions

In summary, our results suggest that moose aim to reach a certain balance between macronutrients, which is in line with the nutrient balancing hypothesis. We have also shown that the moose in our study attempted to compensate for an imbalanced nutritional state by increasing their intake of browse, the most nutritionally balanced items available to them during the experiment. Our results therefore suggest that although digestible energy is an important factor in herbivore diet selection and a rational choice of currency for many types of studies, using digestible energy alone as a measure obscures the relative importance of the macronutrients to these animals. While foraging decisions can be predicted by reducing costs and benefits of foraging into a single best currency, such as total digestible energy [19–22, 88], the associated term energy maximization is often taken out of this context to describe the actual food choice of ungulate species regardless of circumstance. In our experience this appears to be having a diverse array of potentially adverse implications, including the mistaken notion that captive ungulates should be fed energy-dense diets, a practice that appears to result in ill health and short longevity [83, 89–91]; and the provision of starch- and sugar rich concentrates as supplementary food to free-ranging ungulates [92], which may result in increased forest damage due to compensatory feeding. More research is needed to test how the interactions we have identified in this study play out in the landscape and how this knowledge can be applied in the management of game and forests.

## Supporting Information

S1 File Supplementary methods. (DOCX)

S2 File Supplementary results. (DOCX)

S3 File Supplementary figures. (DOCX)
