# Supplementary material for: The Nutritional Balancing Act of a Large Herbivore: An Experiment with Captive Moose (Alces alces L)
Source: PLoS One. 2016 Mar 17;11(3):e0150870. doi: 10.1371/journal.pone.0150870 (PMC4795764; doi:10.1371/journal.pone.0150870)
Supplement: S1 File — (DOCX) [file pone.0150870.s001.docx]

# Supplementary Information

## S1 File: Supplementary methods

**Table A.** **Feed used in the experiment.** Producer’s list of ingredients and nutrient concentrations (estimations based on ingredients’ known concentrations, which we used to design the pellets) of the two pellet types used in this study (L and H) and the zoo’s regular moose pellet Renfor for comparison. Lantmännen Lantbruk produced these pellets. The nutrient estimations listed were used to design the pellet types. For nutrient estimations based on our own chemical analyses, which we used for all intake calculations, see Table 2, main text.

|  | % of ww | | |
| --- | --- | --- | --- |
| Ingredient | **Low-protein (L)** | **High-protein (H)** | **Renfor** |
| Barley | 12.8 | 6.3 | 15.0 |
| Beet (whole) molasses | 4.0 | 4.0 | 4.0 |
| Beet pulp with molasses | 8.2 | 0.0 | 8.6 |
| Beet pulp without molasses | 30.0 | 0.0 | 16.2 |
| Oats | 15.2 | 10.0 | 0.0 |
| Oats peel | 25.0 | 10.0 | 25.0 |
| Palm kernel expeller meal | 0.3 | 10.0 | 5.0 |
| Soya (46% Cp) | 0.0 | 31.0 | 0.0 |
| Wheat | 0.0 | 14.2 | 3.6 |
| Wheat bran | 0.9 | 9.0 | 15.0 |
| Distiller's dry grain | 0.00 | 0.00 | 4.32 |
| Fatty acid mixture | 0.85 | 0.50 | 0.59 |
| Lignosulphonate (bond) | 0.00 | 0.50 | 0.00 |
| Lime | 1.41 | 2.20 | 2.00 |
| Monocalciumphosphate | 0.76 | 0.80 | 0.03 |
| Rock salt | 0.30 | 0.50 | 0.30 |
| Vitamins and trace elements | 0.30 | 0.97 | 0.30 |
|  |  | | |
| Nutrient | **% of dm** | | |
| Crude protein | 9.9 | 25.0 | 14.1 |
| Crude fat | 3.3 | 3.8 | 3.7 |
| Ash | 7.2 | 9.2 | 8.7 |
| NDF | 37.2 | 29.5 | 44.6 |
| TNC | 25.2 | 11.2 | 22.9 |
| ADIN | 8.5 | 6.0 | 12.0 |
| Ca | 11.3 | 14.4 | 12.9 |
| P | 4.0 | 7.2 | 4.5 |
| Mg | 2.8 | 3.6 | 3.9 |
| K | 6.9 | 12.2 | 10.0 |
| Na | 2.1 | 2.7 | 2.3 |

**Housing of study subjects**

The study took place at the Skåne Zoo, certified by the Swedish Board of Agriculture to conduct research within their premises. An ethics permit for the study was granted separately by the Swedish Board of Agriculture (permit no. M 247-11). The study was carried out in two pre-established moose enclosures (3.5 ha and 4 ha) that contained a mature forest canopy for shade and rest, but almost no natural browse. Each enclosure could be divided into two subsections for safe entry by researchers and staff. For a description of feeding arrangements, see “Experimental Procedure”, Methods, main text. All moose were born in captivity. They were in good health as regularly confirmed by the zoo veterinarian. The normal care of the moose included the twice daily provision of newly harvested branches of broadleaved trees. However, the vast majority of the food provided consisted of pellets. There were no structural changes made to the animal’s housing for the sake of this experiment. No moose showed signs of ill health during the entire period.

**Experimental procedure**

For nutritional analyses we collected plant samples that were consistent with the item type and bite sizes observed to be eaten. In conjunction with supplying browse each morning and afternoon, a ninth branch was randomly selected as an unbrowsed representative of each batch. The branch was weighed, twigs were counted and all the twigs under a certain diameter (6 mm for *S. caprea* and *S.* *cinerea;* 9 mm for *S. fragilis*) were removed from the branch, weighed and placed in plastic bags. Decisions regarding maximum diameters were guided by the observations described in the main text. We removed bark in strips of a width and length similar to how the moose removed bark (1-2 cm wide, 5-20 cm long). In total 51 branches of *S. caprea* were sampled, 18 of *S. cinerea* and 39 of *S. fragilis*.

**Chemical Analysis**

Because the diameter of twigs greatly influences the degree of digestibility ([Hjeljord et al. 1982](#_ENREF_4), [Vivas et al. 1991](#_ENREF_14)), we divided the defrosted twigs into two categories: “fine” (<4 mm diam. for *S. caprea* and *S. cinerea*; <3 mm for *S. fragilis*) and “coarse” (4-6 mm diam. for *S. caprea* and *S. cinerea*; 3-9 mm for *S. fragilis*). The cut-off diameter between “fine” and “coarse” twig depended on the tree species’ general twig anatomy which was similar between *S. caprea* and *S. cinerea* but differed for *S. fragilis*, which has longer, more slender shoots.

Residual moisture in all ground material was determined by oven drying at 103 °C for 16 h. Ash was determined by ignition at 550 °C for 3 h. Nitrogen concentration (totN) was determined by the Kjeldahl method ([NCFA 1976](#_ENREF_2)) using a 2020 Digestor and a 2400 Kjeltec Analyser Unit (FOSS Analytical A/S, Hillerød, Denmark). Crude fat content was estimated by petroleum spirit extraction (bp 40-60°C) following hydrolysis in 3N HCl ([Anon. 1984](#_ENREF_3)) using a 1047 Hydrolyzing Unit and a Soxtec System HT 1043 Extraction Unit (FOSS Analytical A/S). Because various non-fat components, such as waxes and cutin, were likely extracted from the twig and bark samples using this method, we subtracted 1 from the result to avoid overestimation of fat in browse items ([Palmquist and Jenkins 2003](#_ENREF_8)).

Tannins may also limit the availability of N for moose ([McArt et al. 2009](#_ENREF_7), [Spalinger et al. 2010](#_ENREF_10)). However, the protein-precipitation capacity of tannins in stems is about half of that in leaves ([*Salix* *scouleriana* and *S. alaxensis,* Spalinger et al. 2010](#_ENREF_10)) and the two grain-based pellet types were assumed to have very low (and similar) tannin concentrations. Therefore, we did not assess the effect of tannins on protein digestibility. The ADIN subtraction method represents protein availability well for ruminants ([Waters et al. 1992](#_ENREF_16), [Van Soest 1994](#_ENREF_11), [Licitra et al. 1996](#_ENREF_6)) and for herbivores in general when tannins are absent ([Wallis et al. 2012](#_ENREF_15)).

**Data Analysis**

Rates of ruminal fermentation, and thus the resultant energetic value of digestible carbohydrates, can be highly variable ([Van Soest et al. 1991](#_ENREF_12), [Allen 1997](#_ENREF_1)). While our measure of digestible fiber incorporates a good estimate of digestibility using *in vitro* techniques with moose rumen liquid, we have assumed total fermentation of non-structural carbohydrates. Our measure of NPE may therefore be an overestimate. However, because we only use this measure in regards to pellets (where fermentation rates should be similar for both pellet types) the introduced error should not be of concern.

**Statistical Analysis**

The GLMM and LMMs were done in the MASS ([Venables and Ripley 2002](#_ENREF_13)) and nlme ([Pinheiro et al. 2013](#_ENREF_9)) packages, respectively, while the post-hoc tests of the LMMs were done by applying the multcomp package ([Hothorn and Westfall 2008](#_ENREF_5)). The statistical models were evaluated concerning homoscedasticity, and other patterns in the residuals, by plotting the Pearson residuals against the predicted values. To achieve homoscedasticity all response variables analyzed by LMMs were log-transformed, with the exception of daily intake of non-protein energy.

## References used in Supplementary Information

Allen, M. S. 1997. Relationship between fermentation acid production in the rumen and the requirement for physically effective fiber. J Dairy Sci **80**:1447-1462.

NCFA. Nitrogen. Determination in food and feed according to Kjeldahl. Nordic Committee on Food Analysis, No. 6, 3rd ed. Esbo, Finland., 1976.

Anon. 1984. Determination of crude oils and fat: Method B. Official J Europ Communities.

Hjeljord, O., F. Sundstol, and H. Haagenrud. 1982. The nutritional value of browse to moose. J Wildl Manag **46**:333-343.

Hothorn, T., and P. Westfall. 2008. Simultaneous Inference in General Parametric Models. Biom Journal **50**:346-363.

Licitra, G., T. M. Hernandez, and P. J. VanSoest. 1996. Standardization of procedures for nitrogen fractionation of ruminant feeds. Anim Feed Sci Techn. **57**:347-358.

McArt, S. H., D. E. Spalinger, W. B. Collins, E. R. Schoen, T. Stevenson, and M. Bucho. 2009. Summer dietary nitrogen availability as a potential bottom-up constraint on moose in south-central Alaska. Ecology **90**:1400-1411.

Palmquist, D. L., and T. C. Jenkins. 2003. Challenges with fats and fatty acid methods. J Anim Sci. **81**:3250-3254.

Pinheiro, J., D. Bates, S. DebRoy, D. Sarkar, and t. R. D. C. Team. 2013. nlme: Linear and nonlinear mixed effects models. R package version 3.1-113.

Spalinger, D. E., W. B. Collins, T. A. Hanley, N. E. Cassara, and A. M. Carnahan. 2010. The impact of tannins on protein, dry matter, and energy digestion in moose (*Alces alces*). Can J Zool. 88 (10):3583-3597.

Van Soest, P. J. 1994. Nutritional ecology of the ruminant. 2nd edition. Cornell Univ Press.

Van Soest, P. J., J. B. Robertson, and B. A. Lewis. 1991. Methods for dietary fiber, neutral detergent fiber, and nonstarch polysaccharides in relation to animal nutrition. J Dairy Sci. **74**:3583-3597.

Venables, W. N., and B. D. Ripley. 2002. Modern applied statistics with S. 4th edition. Springer, New York.

Vivas, H. J., B. E. Saether, and R. Andersen. 1991. Optimal twig size selection of a generalist herbivore, the moose *Alces alces* - implications for plant herbivore interactions. J Anim Ecol. **60**:395-408.

Wallis, I. R., M. J. Edwards, H. Windley, A. K. Krockenberger, A. M. Felton, M. Quenzer, J. U. Ganzhorn, and W. J. Foley. 2012. Food for folivores: nutritional explanations linking diets to population density. Oecologia **169**:281-291.

Waters, C. J., M. A. Kitcherside, and A. J. F. Webster. 1992. Problems associated with estimating the digestibility of undegraded dietary nitrogen from acid-detergents insoluble nitrogen. Anim Feed Sci Techn. **39**:279-291.
