# Supplementary material for: The Nutritional Balancing Act of a Large Herbivore: An Experiment with Captive Moose (Alces alces L)
Source: PLoS One. 2016 Mar 17;11(3):e0150870. doi: 10.1371/journal.pone.0150870 (PMC4795764; doi:10.1371/journal.pone.0150870)
Supplement: S2 File — (DOCX) [file pone.0150870.s002.docx]

## S2 File: Supplementary results

**Table A.** Model parameters and statistics for the three best linear mixed models described in Table 3, main text. The parameters for the first two models show how the intake of pellets and browse (g dm/individual/ W^0.75^/day) differed between the treatments (the five different week-long dietary regimes tested: high protein (H), low protein (L), Buffet 1, Post H buffet, Post L buffet). Parameters for the third model show the relationship between browse intake and browse availability (g edible browse/batch). Results from post-hoc tests are displayed in Table 4, the main text.

| Treatments | Value | Std Error | t-value | p-value |
| --- | --- | --- | --- | --- |
| *Pellet intake (186 obs) LMM 2* | | | | |
| Intercept (H) | α = 4.38 | 0.065 | 67.74 | <0.001 |
| L | β = -0.162 | 0.080 | -2.02 | 0.046 |
| Post H buffet | β = -0.028 | 0.080 | 0.348 | 0.728 |
| Post L buffet | β = -0.037 | 0.080 | -0.466 | 0.642 |
| Buffet 1 | β = 0.055 | 0.086 | 0.637 | 0.525 |
|  | | | | |
| *Browse intake A (186 obs) LMM 4* | | | | |
| Intercept (H) | α = 0.179 | 0.189 | 0.947 | 0.345 |
| L | β = 0.053 | 0.199 | 0.269 | 0.789 |
| Post H buffet | β = -0.524 | 0.199 | -2.642 | 0.009 |
| Post L buffet | β = -0.295 | 0.199 | -1.484 | 0.141 |
| Buffet 1 | β = -0.840 | 0.213 | -3.949 | <0.001 |
|  | | | | |
| *Browse intake B (141 obs) LMM 1* | | | | |
| Intercept | α = -0.408 | 0.349 | -1.167 | 0.247 |
| Browse avail. | β = 0.000 | 0.000 | 0.846 | 0.400 |

**Table B.** Model parameters and statistics for the two linear mixed models testing how adult moose individuals’ daily protein energy intake (PE) and non-protein energy intake (NPE) (MJ/individual/ W^0.75^/day) differed between the treatments (the five different week-long dietary regimes tested: high protein (H), low protein (L), Buffet 1, Post H buffet, Post L buffet). Results from post-hoc tests are displayed in Table 5, the main text.

| Treatments | Value | Std Error | t-value | p-value |
| --- | --- | --- | --- | --- |
| *Protein energy intake (125 obs)* | | | | |
| Intercept (H) | α = -1.153 | 0.073 | -15.86 | <0.001 |
| L | β = -1.148 | 0.095 | -12.15 | <0.001 |
| Post H buffet | β = -0.621 | 0.094 | -6.575 | <0.001 |
| Post L buffet | β = -0.570 | 0.095 | -6.032 | <0.001 |
| Buffet 1 | β = 0.449 | 0.100 | -4.499 | <0.001 |
|  | | | | |
| *Non-protein energy intake (125 obs)* | | | | |
| Intercept (H) | α = 0.789 | 0.064 | 12.36 | <0.001 |
| L | β = 0.109 | 0.070 | 1.560 | 0.121 |
| Post H buffet | β = 0.206 | 0.070 | 2.956 | 0.004 |
| Post L buffet | β = 0.117 | 0.070 | 1.682 | 0.095 |
| Buffet 1 | β = 0.174 | 0.073 | 2.355 | 0.020 |
