# Supplementary material for: The Nutritional Balancing Act of a Large Herbivore: An Experiment with Captive Moose (Alces alces L)
Source: PLoS One. 2016 Mar 17;11(3):e0150870. doi: 10.1371/journal.pone.0150870 (PMC4795764; doi:10.1371/journal.pone.0150870)
Supplement: S3 File — (DOCX) [file pone.0150870.s003.docx]

## S3 File: Supplementary figures

**Figure A.** Mean nutritional balance (protein energy PE: non-protein energy NPE) of the pellet meals ingested by adult moose (A) and calves (B) per 4-hour interval during the first buffet week of the experiment. Their intake ratio PE:NPE stabilised around 30-60 hours from the start of the week (i.e. around interval 8-15). Solid triangle = Cow 1; Cross = Cow 2; solid square = Bull 1; star = Bull 2; solid diamond = Calf 1; empty square = Calf 2.
